# Optimal cutoff scores of the Chinese version of 15-item negative symptom assessment that indicate prominent negative symptoms of schizophrenia

**DOI:** 10.3389/fpsyt.2023.1154459

**Published:** 2023-04-17

**Authors:** Qi Zhou, Cheng-cheng Pu, Bing-jie Huang, Qi Miao, Tian-hang Zhou, Zhang Cheng, Tian-Qi Gao, Chuan Shi, Xin Yu

**Affiliations:** ^1^Peking University Sixth Hospital, Beijing, China; ^2^Peking University Institute of Mental Health, Beijing, China; ^3^National Health Commission Key Laboratory of Mental Health, Peking University, Beijing, China; ^4^National Clinical Research Center for Mental Disorders, Peking University Sixth Hospital, Beijing, China; ^5^Shandong Mental Health Center, Shandong University, Jinan, China

**Keywords:** 15-item negative symptom assessment (NSA-15), cutoff score, receiver operating characteristic curve, prominent negative symptoms, schizophrenia

## Abstract

**Objective:**

The Chinese version of 15-item negative symptom assessment (NSA-15) is an instrument with a three-factor structure specifically validated for assessing negative symptoms of schizophrenia. To provide a reference for future practical applications in the recognition of schizophrenia patients with negative symptoms, this study aimed to determine an appropriate NSA-15 cutoff score regarding negative symptoms to identify prominent negative symptoms (PNS).

**Methods:**

A total of 199 participants with schizophrenia were recruited and divided into the PNS group (*n* = 79) and non-PNS group (*n* = 120) according to scale for assessment of negative symptoms (SANS) scores. Receiver-operating characteristic (ROC) curve analysis was used to determine the optimal NSA-15 cutoff score for identifying PNS.

**Results:**

The optimal cutoff NSA-15 score for identifying PNS was 40. Communication, emotion and motivation factors in the NSA-15 had cutoffs of 13, 6, and 16, respectively. The communication factor score had slightly better discrimination than scores on the other two factors. The discriminant ability of the global rating of the NSA-15 was not as good as that of the NSA-15 total score (area under the curve (AUC): 0.873 vs. 0.944).

**Conclusion:**

The optimal NSA-15 cutoff scores for identifying PNS in schizophrenia were determined in this study. The NSA-15 provides a convenient and easy-to-use assessment for identifying patients with PNS in Chinese clinical situations. The communication factor of the NSA-15 also has excellent discrimination.

## 1. Introduction

In schizophrenia, negative symptoms are a core dimension of symptoms and are closely associated with poor outcomes and functioning (1, 2). A total of 90% of patients experience at least one negative symptom during their first psychotic episode; despite medical treatment, approximately 35%–70% of patients still experience persistent negative symptoms (3, 4). In clinical practice, 61% of outpatients experience one moderate or even severe negative symptom, although they are considered clinically stable (5). In recent decades, negative symptoms have received substantial attention in terms of their conceptualization as well as their relationship with remission and real-life functioning. However, there is still an unmet need for the treatment of this dimension (6). The schizophrenia section of the European psychiatric association (EPA) proposed the assessment of negative symptoms in clinical trials and practice. Prominent negative symptoms (PNS) have been widely used to evaluate the efficacy of drugs (7), as the persistence of negative symptoms over time has not been evaluated, similar to in clinical practice.

To date, researchers have used different assessment tools with different criteria to identify PNS (8–10). PNS cutoff scores have been identified using several negative symptom scales, such as the positive and negative syndrome scale (PANSS) (9), the scale for the assessment of negative symptoms (SANS) (10) and the clinical assessment interview for negative symptoms (CAINS) (11).

However, the PANSS (12) is designed to evaluate global symptoms, including positive symptoms, not specifically negative symptoms. Both the negative symptom subscale of the PANSS and SANS (13) consist of cognitive symptoms, which can be categorized into other dimensions, indicating conceptual and methodological limitations. Compared to the abovementioned scales, the Negative Symptom Assessment-16 (NSA-16) has better content validity, as it specifically assesses negative symptoms. The CAINS is also a newer instrument designed to overcome the limitations of older instruments, but it has been reported to have low convergent validity (14).

The NSA-16 examines the presence, severity and range of negative symptoms of schizophrenia (15). The Chinese version of the NSA, which is recommended to include 15 items, has been validated (16). The NSA-15 shows excellent convergent validity and good divergent validity with a three-factor structure consisting of communication, emotion and motivation factors (16). It is an explicit tool with well-defined items and detailed rating criteria, which is easy for clinical raters to use. Indeed, the consistency may be better for different raters and centers as it is a standardized semi-structured interview. The Chinese version of the NSA-15 is a classic assessment tool with good validity in the Chinese setting for assessing negative symptoms of schizophrenia. Additionally, the NSA-15 includes a global rating that evaluates the negative symptoms as a whole. However, the cutoff values for identifying PNS remain unknown. This study aimed to determine the optimal NSA-15 cutoff score for identifying PNS in schizophrenia and to confirm the discriminative ability of the NSA-15 and its three factors.

## 2. Materials and methods

### 2.1. Participants

This was a cross-sectional study, and participants were recruited from the Peking University Sixth Hospital in Beijing. The inclusion criteria were as follows: met the criteria for schizophrenia from the Diagnostic and Statistical Manual of Mental Disorders, fourth edition (DSM-IV) and aged between 16 and 60 years. The exclusion criteria were as follows: comorbidities consisting of other DSM-IV Axis I disorders; a history of head injury or neurological disorder; severe or unstable somatic disease; pregnancy or breastfeeding; and modified electroconvulsive therapy received in the previous 3 months. Diagnoses were made by experienced psychiatrists using the Structured Clinical Interview for DSM-IV Disorders (SCID-I) (17). This study was approved by the Ethics Committees of the Institute of Psychology and the Institute of Mental Health (Peking University Sixth Hospital). All participants provided written informed consent; if the participant was under the age of 18 years, a parent or legal guardian provided written informed consent.

A total of 199 schizophrenia patients participated in this study. The average age was 29.31 years (standard deviation (SD) = 9.99), and the mean years of education was 13.46 years (SD = 2.61). A total of 51.8% of the patients were male.

### 2.2. Measures

#### 2.2.1. The Chinese version of NSA-15

The original NSA (15) contains 16 items, each of which is rated on a six-point Likert scale; higher scores reflect more severe negative symptoms. At the end of the scale, there is a global negative symptom rating based on the interviewer’s impression of subject’s negative symptoms. It is a semi-structured interview and takes approximately 15–30 min to complete.

A Chinese version of NSA was developed. Psychometric analysis indicated that the original five-factor model of the NSA-16 was not suitable and suggested a three-factor structure (communication, emotion and motivation) consisting of 15 items which termed NSA-15 (16). The item 6 of the original NSA-16 which reflects the affect modulation was excluded from the scale. In this study, we used the Chinese version of NSA-15, which has been validated in the Chinese context.

#### 2.2.2. Other clinical assessments

The SANS (13) was used to comprehensively and specifically assess participants’ negative symptoms and determine PNS based on an established method. The SANS consists of five dimensions of negatives symptoms, and each dimension includes several independent items and a global item. Every global item generally reflects the severity of the corresponding dimension. The SANS summary score is the sum of 5 global items of SANS, ranging from 0 to 25. The SANS summary score equal to or greater than 10 was used to define patients with PNS (18, 19). The PANSS (12, 20) was used to evaluate overall symptoms, including positive, negative and general psychopathology symptoms of schizophrenia. We used the Calgary depression scale for schizophrenia (CDSS) (21) and rating scale for extrapyramidal side effects (RSESE) (22) to assess depressive symptoms and extrapyramidal side effects. Social functioning was assessed using the Personal and Social Performance Scale (PSP) (23); higher scores on this scale indicate better social functioning.

### 2.3. Statistical analysis

Data were analyzed using SPSS 24.0 and MedCalc 20.0. Statistical tests used a two-tailed 5% significance threshold for all data analyzed. First, participants were divided into the PNS and non-PNS groups according to the SANS summary scores. Independent-sample *t*-tests, Mann–Whitney *U*-tests or chi-square tests, as appropriate, were used to compare demographic and clinical features between the PNS group (*n* = 79) and the non-PNS group (*n* = 120). Then, receiver-operating characteristic (ROC) curve analysis was performed to calculate the appropriate cutoff scores of the NSA-15 and its three factors as well as the corresponding sensitivity and specificity. When relevant, 95% confidence intervals (CIs) were calculated. Next, we calculated the area under the curve (AUC) to evaluate the discriminatory ability of the NSA-15. An AUC of 0.7–0.79 was defined as acceptable discrimination, an AUC of 0.8–0.89 was defined as excellent discrimination, and an AUC of 0.9–1.0 was defined as outstanding discrimination (24). We used the *z*-test to compare the AUC values of the NSA-15, its three factors and the global symptom rating.

## 3. Results

### 3.1. Demographic and clinical features of participants

Table 1 shows the detailed data of the 199 schizophrenia patients. Based on SANS summary scores, 79 patients were placed in the PNS group. The prevalence of PNS was 39.7% according to the SANS. Group comparisons showed that patients in the PNS group were younger and had a shorter duration of illness than those in the non-PNS group (*p* < 0.05). In addition, the patients with PNS had more severe positive, negative and general psychopathology symptoms according to the PANSS and more severe extrapyramidal side effects according to the RSESE. Scores of communication, emotion and motivation indicating different dimensions of negative symptoms were all higher in patients of PNS group. The PNS group also had worse functioning according to the PSP (*p* < 0.05). The two groups showed no significant differences in gender ratio, years of education and depressive symptoms.

**Table 1:** Demographic and clinical information for patients with and without PNS.

	PNS (*n* = 79)	non-PNS (*n* = 120)	Total participants (*n* = 199)	*Z*/*t*/*χ*^2^	*P*
Age (years)	27.58 ± 10.52	30.45 ± 9.50	29.31 ± 9.99	−1.996	0.047
Sex (male, %)	43(54.43%)	60(50.00%)	103(51.76%)	−0.374	0.541
Education (years)	13.04 ± 2.47	13.73 ± 2.66	13.46 ± 2.61	−1.854	0.065
PANSS-total	70.99 ± 16.95	49.38 ± 13.53	57.96 ± 18.32	9.955	<0.001
PANSS-P	15.22 ± 6.41	12.26 ± 5.36	13.43 ± 5.96	3.519	0.001
PANSS-N	23.76 ± 5.71	12.48 ± 4.41	16.95 ± 7.43	14.874	<0.001
PANSS-G	32.01 ± 9.54	24.77 ± 6.59	27.64 ± 8.64	5.88	<0.001
NSA-15 total	51.51 ± 10.41	29.92 ± 8.84	38.49 ± 14.21	15.698	<0.001
NSA-Communication	19.18 ± 5.67	9.76 ± 2.96	13.50 ± 6.26	12.094	<0.001
NSA-emotion	8.44 ± 1.65	5.52 ± 2.67	6.68 ± 2.72	9.550	<0.001
NSA-motivation	23.89 ± 5.02	14.64 ± 5.44	18.31 ± 6.95	12.294	<0.001
NSA-global rating	4(4–5)	3(2–3.75)	3(3–4)	9.185	<0.001
RSESE	0(0–1)	0(0–1)	0(0–1)	2.146	0.032
CDSS	0(0–0)	0(0–1)	0(0–1)	0.803	0.422
PSP	42.33 ± 14.72	60.11 ± 15.04	53.05 ± 17.24	−8.229	<0.001

PANSS, positive and negative syndrome scale (PANSS); PANSS-N, negative symptoms subscale of the PANSS; PANSS-P, positive symptoms subscale of the PANSS; PANSS-G, general pathology subscale of the PANSS; SANS, scale for the assessment of negative symptoms; NSA-15, 15-item negative symptom assessment; RSESE, rating scale for extrapyramidal side effects; CDSS, Calgary depression scale for schizophrenia; PSP, personal and social performance scale; PNS, prominent negative symptoms.

### 3.2. Cutoff scores, sensitivity and specificity of the NSA-15

Table 2 presents the cutoff scores for the NSA-15, its three factors and the global rating with the corresponding sensitivity and specificity values. The cutoff score for the NSA-15 (ranging from 37 to 44) showed high sensitivity (92.41 to 73.42%) and specificity (80.83 to 93.33%). We used the maximum Youden’s index value to determine the optimum cutoff value for diagnostic tests that provided a numerical result. Based on the ROC curve analysis, the NSA-15 total score cutoff with the maximum Youden’s index used to identify PNS was 40. Furthermore, the cutoff score of the communication factor ranged from 11 to 15 and showed high sensitivity (92.41–72.15%) and specificity (71.67–95%). The cutoff score of the emotion factor ranged from 5 to 7 with high sensitivity (97.47–75.95%) and moderate specificity (45.83–66.67%). The cutoff score of the motivation factor ranged from 15 to 18 with high sensitivity (98.73–82.28%) and moderate specificity (60–78.33%). According to the ROC curve analysis, the best cutoff values of the three factors (communication, emotion and motivation) were 13, 6, and 16, respectively. The cutoff value of the NSA-15 global rating was three.

**Table 2:** The cutoff scores and corresponding sensitivity and specificity of the negative symptom assessment (NSA-15).

Criterion	Sensitivity	95% CI	Specificity	95% CI	+LR	−LR
NSA-15 total
≥15	100	95.4–100.0	0	0.0–3.0	1	-
>35	96.20	89.3–99.2	74.17	65.4–81.7	3.72	0.051
>36	93.67	85.8–97.9	79.17	70.8–86.0	4.5	0.08
>37	92.41	84.2–97.2	80.83	72.6–87.4	4.82	0.094
>38	86.08	76.5–92.8	85.00	77.3–90.9	5.74	0.16
>39	84.81	75.0–91.9	88.33	81.2–93.5	7.27	0.17
***>40***	***83.54***	***73.5–90.9***	***90.00***	***83.2–94.7***	***8.35***	***0.18***
>41	81.01	70.6–89.0	90.83	84.2–95.3	8.84	0.21
>42	79.75	69.2–88.0	91.67	85.2–95.9	9.57	0.22
>43	77.22	66.4–85.9	92.50	86.2–96.5	10.3	0.25
>44	73.42	62.3–82.7	93.33	87.3–97.1	11.01	0.28
>45	68.35	56.9–78.4	93.33	87.3–97.1	10.25	0.34
>74	0	0.0–4.6	100.00	97.0–100.0	-	1
Communication
≥7	100	95.4–100.0	0	0.0–3.0	1	-
>8	98.73	93.1–100.0	45.83	36.7–55.2	1.82	0.028
>9	96.20	89.3–99.2	56.67	47.3–65.7	2.22	0.067
>10	96.20	89.3–99.2	66.67	57.5–75.0	2.89	0.057
>11	92.41	84.2–97.2	71.67	62.7–79.5	3.26	0.11
>12	89.87	81.0–95.5	78.33	69.9–85.3	4.15	0.13
***>13***	***83.54***	***73.5–90.9***	***86.67***	***79.3–92.2***	***6.27***	***0.19***
>14	74.68	63.6–83.8	92.50	86.2–96.5	9.96	0.27
>15	72.15	60.9–81.7	95.00	89.4–98.1	14.43	0.29
>16	64.56	53.0–75.0	97.50	92.9–99.5	25.82	0.36
>17	54.43	42.8–65.7	99.17	95.4–100.0	65.32	0.46
>33	0	0.0–4.6	100	97.0–100.0	-	1
Emotion
≥2	100	95.4–100.0	0	0.0–3.0	1	-
>4	98.73	93.1–100.0	35.83	27.3–45.1	1.54	0.035
>5	97.47	91.2–99.7	45.83	36.7–55.2	1.80	0.055
***>6***	***84.81***	***75.0–91.9***	***66.67***	***57.5–75.0***	***2.54***	***0.23***
>7	75.95	65.0–84.9	72.50	63.6–80.3	2.76	0.33
>8	46.84	35.5–58.4	86.67	79.3–92.2	3.51	0.61
>9	26.58	17.3–37.7	90.00	83.2–94.7	2.66	0.82
>12	0	0.0–4.6	100.00	97.0–100.0	-	1
Motivation
≥6	100	95.4–100.0	0	0.0–3.0	1	-
>14	98.73	93.1–100.0	50.83	41.6–60.1	2.01	0.025
>15	98.73	93.1–100.0	60.00	50.7–68.8	2.47	0.021
***>16***	***96.20***	***89.3–99.2***	***67.50***	***58.3–75.8***	***2.96***	***0.056***
>17	89.87	81.0–95.5	72.50	63.6–80.3	3.27	0.14
>18	82.28	72.1–90.0	78.33	69.9–85.3	3.8	0.23
>19	77.22	66.4–85.9	81.67	73.6–88.1	4.21	0.28
>20	70.89	59.6–80.6	87.50	80.2–92.8	5.67	0.33
>21	63.29	51.7–73.9	88.33	81.2–93.5	5.42	0.42
>35	0.00	0.0–4.6	100.00	97.0–100.0	-	1
Global rating
>2	100.00	95.4–100.0	32.50	24.2–41.7	1.48	0.00
***>3***	***86.08***	***76.5–92.8***	***75.00***	***66.3–82.5***	***3.44***	***0.19***
>4	44.30	33.1–55.9	97.50	92.9–99.5	17.72	0.57
>5	15.19	8.1–25.0	99.17	95.4–100.0	18.23	0.86
>6	2.53	0.3–8.8	100.00	97.0–100.0	-	0.97
>7	0	0.0–4.6	100.00	97.0–100	-	1

CI, confidence interval; +LR, positive likelihood ratio; −LR, negative likelihood ratio. The bold and italicized rows indicate the suggested cutoff scores.

### 3.3. Diagnostic accuracy comparison between the NSA-15 total score and its three factors

**Figure 1:**
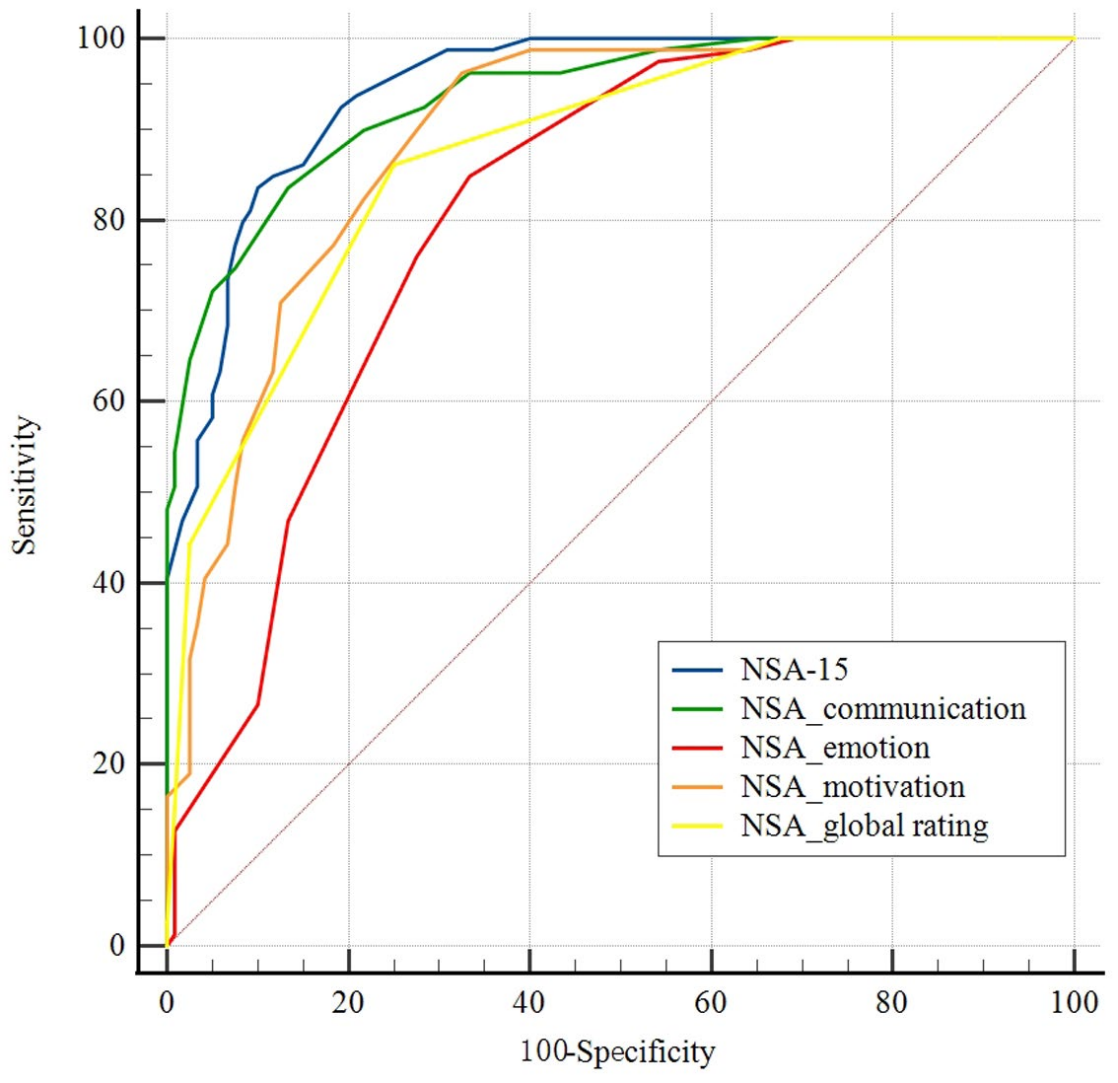
Receiver-operating characteristic (ROC) curves of cutoff scores on the negative symptom assessment (NSA-15), its three factors and the global rating. Blue: the NSA-15; green: NSA-15 communication factor; red: NSA-15 emotion factor; orange: NSA-15 motivation factor; and yellow: NSA-15 global rating.

Figure 1 shows the diagnostic accuracy of the NSA-15 total score as well as its three factors and the global rating. The AUC values were 0.944 (95% CI: 0.916 to 0.972) and 0.935 (95% CI: 0.903 to 0.968) for the NSA-15 total score and the communication factor, indicating outstanding discriminatory power. The AUC values of the emotion factor and motivation score were 0.810 (95% CI: 0.752 to 0.869) and 0.891 (95% CI: 0.848 to 0.935), respectively, demonstrating excellent discriminatory power. In addition, the AUC of the NSA-15 global rating was 0.873 (95% CI: 0.818–0.916).

There was no significant difference in AUC between the NSA-15 total score and the communication factor according to pairwise comparisons (*z* = 0.673, *p* = 0.501). The positive predictive value (PPV) and negative predictive value (NPV) of the NSA-15 total score were 84.6 and 89.3%, respectively, and those of the communication factor were 80.5 and 88.9%. The AUC values of the global rating and the motivation factor were both smaller than that of the NSA-15 total score (global rating vs. total score: *z* = 3.959, *p* < 0.001; motivation factor vs. total score: *z* = 3.839, *p* < 0.001). The emotion factor had the worst discriminant ability, with an AUC significantly lower than that of NSA-15 total score, the other two subscales, and the global rating (emotion factor vs. total score: *z* = 5.613, *p* < 0.001; emotion factor vs. communication factor: *z* = 4.191, *p* < 0.001; emotion factor vs. motivation factor: *z* = 2.851, *p* = 0.004; emotion factor vs. global rating: *z* = 2.271, *p* = 0.023).

## 4. Discussion

This study aimed to discover the optimal cutoff scores of the Chinese version of NSA-15 for identifying PNS in schizophrenia. Our results suggested that the optimal cutoff value of the NSA-15 total score for identifying PNS was 40. The cutoff scores for the communication, emotion, and motivation factors were 13, 6, and 16, respectively. The NSA-15 total score and communication factor demonstrated outstanding discriminatory power, better than that of the emotion and motivation factors and that of the global rating. Thus, the NSA-15 may be a robust tool for identifying patients with PNS.

In our study, the prevalence of PNS identified by the NSA-15 was 39.7%, which is close to the estimates reported in recent studies (3, 4). The patients with PNS in our study were younger indicating that negative symptoms may appear early in the course of disease and merit attention. Moreover, patients with PNS also had more severe positive and general psychopathology symptoms as well as extrapyramidal side effects in this study, which indicates that some patients may have experienced PNS due to other symptoms or side effects of medication. Some secondary negative symptoms may not disappear with regular treatment (1); thus, they should not be neglected in clinical practice. It is reported that depression is closely associated with negative symptoms (25), however, there was no difference between patients with and without PNS in this study, which helped rule out the influence of the depressive symptoms. Further studies should recruit more patients with PNS and follow patients to assess the stability of negative symptoms.

Our results indicated that the NSA-15 is an appropriate assessment tool for identifying schizophrenia patients with PNS, which could facilitate associated research in the future. The cutoff scores of the NSA-15 total score ranged from 37 to 44. As sensitivity and specificity may vary and sensitivity is inversely related to specificity (26), we recommend using a cutoff score >40 (NSA-15 total score) or >13 (communication factor) to identify PNS in Chinese patients with schizophrenia, as these scores had the highest Youden’s index. Although there was no significant difference in AUC values between the NSA-15 total score and the communication factor, combined with the results of the PPV and NPV of the total score and communication factor, in general, the NSA-15 total score has the highest discriminatory power for identifying PNS in schizophrenia. Additionally, the NSA-15 total score consists of multifaceted information, it may be better to identify PNS using total score of the NSA-15. The NSA-15 global rating combines the relatively objective items with the rater’s subjective impressions and judgments. Our results showed that the discriminatory power of the global rating was lower than that of the total score, which further emphasizes the importance of concrete and detailed assessments of negative symptoms.

The Spanish version of NSA suggested cutoff scores of 31 to identify negative symptoms (27). The Spanish version used the Clinical Global Impression (CGI) scale as the discriminant standard and the different tools used may be responsible for the difference. The criterion of CGI was to identify schizophrenia patients with negative symptoms and not specifically assess the negative symptoms. The CGI rating has been proved to have weak association with negative symptoms assessed by SANS, ae well as negative symptom subscale of PANSS (28), so this may explain the smaller cutoff scores of the Spanish version. What’s more, expression emotion and communication deficits vary by ethnicity (29), the discrepancy between our results and those of a previous report (27) on the suitable NSA-15 cutoff score may be due to cultural differences and further highlights the importance of determining culture-specific cutoff scores.

Negative symptoms consist of various dimensions, including alogia, blunted affect, anhedonia, asociality and avolition (30). Compared to traditional assessment tools, such as the SANS and PANSS, the NSA-15 does not encompass symptoms from other dimensions not belonging to negative symptoms. NSA-15 specifically assesses the negative symptoms and is comprised of multiple dimensions covering communication, emotion and motivation. As negative symptoms change with the trajectory of the clinical progress (31), the characteristic with involvement and distinction of multiple dimensions of negative symptoms may improve the sensitivity of the NSA-15 for individual differences and different stages of schizophrenia.

In addition, we found that among the three NSA-15 factors, the communication factor provided better discriminatory power than the emotion and motivation factors. Currently, the conceptualization of negative symptoms encompasses two distinct subdomains: diminished expression and avolition-apathy, both of which are associated with impaired real-world functioning after controlling for cognition and functional capacity (32); avolition-apathy seems to have greater impacts than diminished expression (33). The communication factor assesses participants’ explicit performance (and impairment) of body language and verbal expressions. The emotion factor measures emotional range and display, and the motivation factor reflects social drive and personal interest and purpose. Our findings suggest that deficits in communication may be more strongly linked to PNS, in contrast to a previous study (11), which suggested that hedonic capacity and motivation are more closely associated with negative symptoms. The communication factor consists of both verbal and non-verbal expression. A meta analysis (34) indicates that facial emotion recognition impairment, which is part of non-verbal expression, is a stable feature in schizophrenia. In this regard, the communication factor could provide relatively more objective information from the faces and behaviors and may better reflect the general state of the participant. The reduction of communication has been turned to reflect the overall deficits in the ability to communicate with others, and have complex interactions with cognition, emotion and verbal function (35, 36). As a result, the decrease in communication directly influences the social participation and interaction, as well as the normal social relationships. On the other hand, the experiential deficits of negative symptoms, such as amotivation, will lead to the decreased goal-directed words and behaviors, which are also embodied in the assessment of expression dimension. So this indicates that communication factor may better reflect the general negative symptoms and is closely associated with PNS. As secondary negative symptoms were not ruled out in this study, the differences of clinical characteristics may contribute to the difference in the results. Differences in the instruments used may partly explain this inconsistency. Negative symptoms are heterogeneous, with ambiguous boundaries and potential joint effects. To date, the majority of research on the dimensions of negative symptoms has focused on the experiential dimension, including motivation and interest (37, 38). The close relationship between the NSA-15 total score and the communication factor indicates that the expressive dimension also plays an important role in negative symptoms and warrants further research. In general, the relationships between the three factors and negative symptoms need further exploration.

There are several limitations to this study. This was a cross-sectional study, and longitudinal changes in symptoms among the recruited patients were not evaluated. Further studies should examine changes in negative symptoms in the PNS and non-PNS groups. Due to the heterogeneity of negative symptoms of schizophrenia, the characteristics of patients in different stages of illness should be explored. In addition, we did not compare important psychometric characteristics of the NSA-15 with those of new generation assessment tools, such as the CAINS, which has been demonstrated to be valid and robust in identifying PNS in schizophrenia (11).

## 5. Conclusion

In summary, the Chinese version of the NSA-15 is an effective assessment tool for identifying PNS in schizophrenia patients. The cutoff point of the NSA-15 total score that identifies PNS in the Chinese setting (score > 40) is recommended for future research and practical applications. The communication factor of the NSA-15 also has excellent discrimination.

## Data availability statement

The raw data supporting the conclusions of this article will be made available by the authors, without undue reservation.

## Ethics statement

The studies involving human participants were reviewed and approved by Ethics Committee of Peking University Sixth Hospital. Written informed consent to participate in this study was provided by the participants’ legal guardian/next of kin.

## Author contributions

XY, CS, and C-cP conceived and designed the study, obtained funding, and supervised the study. QZ and C-cP performed the data analysis and interpretation. QZ, C-cP, and XY conducted the research design and manuscript writing. B-jH, QM, T-hZ, ZC, and T-QG participated in the collection of data. All authors contributed to the article and approved the final submitted version.

## Funding

This study was supported by the National Science Fund China (No. 82171500), the Key Program of Beijing Science and Technology Commission (No. D171100007017002), and the Capital’s Funds for Health Improvement and Research (CRF, No. 2018–4-4116).

## Conflict of interest

The authors declare that the research was conducted in the absence of any commercial or financial relationships that could be construed as a potential conflict of interest.

## Publisher’s note

All claims expressed in this article are solely those of the authors and do not necessarily represent those of their affiliated organizations, or those of the publisher, the editors and the reviewers. Any product that may be evaluated in this article, or claim that may be made by its manufacturer, is not guaranteed or endorsed by the publisher.
